# Genetic homogeneity among *Leishmania* (*Leishmania*) *infantum* isolates from dog and human samples in Belo Horizonte Metropolitan Area (BHMA), Minas Gerais, Brazil

**DOI:** 10.1186/s13071-015-0837-y

**Published:** 2015-04-15

**Authors:** Thais Almeida Marques da Silva, Luciana Inácia Gomes, Edward Oliveira, Wendel Coura-Vital, Letícia de Azevedo Silva, Fabiano Sviatopolk-Mirsky Pais, Henrique Gama Ker, Alexandre Barbosa Reis, Ana Rabello, Mariangela Carneiro

**Affiliations:** Laboratório de Pesquisas Clínicas, Centro de Pesquisas René Rachou, Fundação Oswaldo Cruz, Belo Horizonte, Minas Gerais Brasil; Laboratório de Epidemiologia das Doenças Infecciosas e Parasitárias, Departamento de Parasitologia, Instituto de Ciências Biológicas, Universidade Federal de Minas Gerais, Belo Horizonte, Minas Gerais Brasil; Pós-graduação em Ciências da Saúde: Infectologia e Medicina Tropical, Faculdade de Medicina, Universidade Federal de Minas Gerais, Belo Horizonte, Minas Gerais Brasil; Laboratório de Pesquisas Clínicas, Escola de Farmácia, Universidade Federal de Ouro Preto, Ouro Preto, Minas Gerais Brasil; Grupo de Genômica e Biologia Computacional, Centro de Pesquisas René Rachou, Fundação Oswaldo Cruz, Belo Horizonte, Minas Gerais Brasil; Laboratório de Toxoplasmose, Departamento de Parasitologia, Instituto de Ciências Biológicas, Universidade Federal de Minas Gerais, Belo Horizonte, Minas Gerais Brasil

**Keywords:** *Leishmania* (*Leishmania*) *infantum*, Intraspecific genetic variability, PCR-RFLP

## Abstract

**Background:**

Certain municipalities in the Belo Horizonte Metropolitan Area (BHMA), Minas Gerais, Brazil, have the highest human visceral leishmaniasis (VL) mortality rates in the country and also demonstrate high canine seropositivity. In Brazil, the etiologic agent of VL is *Leishmania* (*Leishmania*) *infantum*. The aim of this study was to evaluate the intraspecific genetic variability of parasites from humans and from dogs with different clinical forms of VL in five municipalities of BHMA using PCR-RFLP and two target genes: kinetoplast DNA (kDNA) and *gp63*.

**Methods:**

In total, 45 samples of DNA extracted from clinical samples (n = 35) or *L. infantum* culture (n = 10) were evaluated. These samples originated from three groups: adults (with or without *Leishmania*/HIV co-infection; n = 14), children (n = 18) and dogs (n = 13). The samples were amplified for the kDNA target using the MC1 and MC2 primers (447 bp), while the Sg1 and Sg2 (1330 bp) primers were used for the *gp63* glycoprotein target gene.

**Results:**

The restriction enzyme patterns of all the samples tested were monomorphic.

**Conclusions:**

These findings reveal a high degree of genetic homogeneity for the evaluated gene targets among *L. infantum* samples isolated from different hosts and representing different clinical forms of VL in the municipalities of BHMA studied.

## Background

Leishmaniasis is among the six endemic diseases considered to be priority diseases worldwide and is characterized by its great diversity and complexity [1]. Approximately 1.3 million new cases of leishmaniasis are estimated to occur annually, and of these cases, 300,000 correspond to visceral leishmaniasis (VL). VL is considered the most severe form of the disease, and it is potentially fatal if left untreated. Between 20,000 and 40,000 people are estimated to die of VL worldwide annually [2,3].

In Brazil, VL is caused by *Leishmania* (*Leishmania*) *infantum* [synonym of *Leishmania* (*Leishmania*) *chagasi*], which is an obligate intracellular protozoan belonging to the *Leishmania donovani* complex [4]. In the urban context, domestic dogs are the main reservoir of this parasite, and the sandfly *Lutzomyia* (*Lutzomyia*) *longipalpis* is the main vector of the disease [5].

Between 2005 and 2011, the mean incidence rate of VL in Brazil was approximately 2.0 cases per 100,000 inhabitants, and the mortality rate was 6.7% [6]. Certain municipalities in the Belo Horizonte Metropolitan Area (BHMA) have had high mortality rates, including Belo Horizonte, where rates ranged from 8.2% in 2007 to 21.8% in 2012, with a mean rate of 17.1% [7]. In several studies conducted in BHMA municipalities, *L. infantum* infection rates ranged from 2.4% to 18.1%, depending on the population studied or the diagnostic technique used [8-10].

Several studies have been conducted to examine the intraspecific genetic variability between *L. infantum* strains and their possible association with different types of hosts [11,12], different geographical origins [13-15], parasite transmission dynamics [16] and the spread of the disease [16-19]. For these purposes, various molecular approaches have been used: (i) randomly amplified polymorphic DNA (RAPD) [14,20,21]; (ii) analysis of DNA regions using multilocus microsatellite typing (MLMT) [13,22]; (iii) simple sequence repeat (SSR)-PCR [14,23]; (iv) analysis using DNA hybridization probes (Southern blotting) [24] and (v) restriction fragment length polymorphism (RFLP) analysis [11,25-28]. Although the BHMA comprises municipalities with high human mortality rates, asymptomatic infection and canine prevalence, few studies have been conducted in this area to evaluate whether genetic variability exists among isolates from different hosts and clinical forms of VL [12,29]. Consequently, the aim of this study was to evaluate the genetic variability of *L. infantum* in humans (children and adults with or without *Leishmania*/HIV co-infection) and in dogs with different clinical forms of VL (symptomatic, oligosymptomatic and asymptomatic) in BHMA municipalities.

## Methods

### Study samples

Thirty-five clinical samples (whole blood, n = 12; bone marrow aspirate, n = 15; and spleen biopsy, n = 8) and 10 *L. infantum* culture samples from human and canine VL cases were used. The samples originated from three groups: VL-symptomatic children (n = 18), VL-symptomatic adults (n = 14) and dogs with different clinical forms of VL (symptomatic, oligosymptomatic and asymptomatic) (n = 13) (Table 1).

**Table 1:** **Characteristics of the evaluated samples**

**Variables**		**Humam**	**Dogs**
		**n (%)**	**n (%)**
Sex	Female	12 (37.5)	7 (53.8)
	Male	20 (62.5)	6 (46.2)
City	Belo Horizonte	19 (59.4)	13 (100)
	Contagem	5 (15.6)	0
	Ribeirão das Neves	4 (12.5)	0
	Betim	1 (3.1)	0
	Ibirité	3 (9.4)	0
HIV	Positive	10 (31.2)	Not applicable
	Negative	22 (68.8)	Not applicable
Sample	Bone marrow aspirate	15 (46.9)	0
	Culture sample	5 (15.6)	5 (38.5)
	Whole bood	12 (37.5)	0
	Spleen biopsy	0	8 (61.5)
Clinical form	Asymptomatic	0	4 (30.8)
	Oligosymptomatic	0	6 (46.1)
	Symptomatic	32 (100)	3 (23.1)
Collection	LPC/CPqRR	32 (100)	0
	LPC/UFOP	0	13 (100)
Year isolation	2010	1 (3.1)	13 (100)
	2011	20 (62.5)	0
	2012	11 (34.4)	0

LPC/CPqRR, Laboratório de Pesquisas Clínicas, Centro de Pesquisas René Rachou.

LPC/UFOP, Laboratório de Pesquisas Clínicas, Universidade Federal de Ouro Preto.

The sex distribution was equal in the group of children (nine females and nine males), with a mean age of 4.5 years old (SD = 2.2). In the adult group, 11 patients were men, and three were women; the mean age was 38.7 years old (SD = 11). Among the adults, 10 individuals had *Leishmania*/HIV co-infection (Table 1).

The human samples (children and adults) were obtained at the cryobank of the Laboratory of Clinical Research, Centro de Pesquisas René Rachou. The canine samples originated from the cryobank of the Laboratory of Clinical Research, Federal University of OuroPreto.

The 45 evaluated DNA samples were selected from VL cases that occurred between the years 2010 to 2012, in five municipalities in the state of Minas Gerais belonging to the BHMA: Belo Horizonte (n = 32, 19 human cases and 13 canine cases), Betim (n = 1 human case), Contagem (n = 5 human cases), Ibirité (n = 3 human cases) and Ribeirão das Neves (n = 4 human cases). All 45 samples were initially analyzed by the PCR-RFLP molecular technique [30] to characterize the samples as *L. infantum*. In addition to being subjected to molecular analyses, the *Leishmania* culture samples were classified at the species level according to the multi-locus enzyme electrophoresis (MLEE) technique, following the protocol established by Cupolillo *et al.* [31], performed in the *Leishmania* Collection of the Oswaldo Cruz Institute.

### Promastigote culture

*Leishmania infantum* promastigote culture aliquots were isolated by bone marrow aspiration from humans and dogs. These samples were cultured in liver infusion tryptose (LIT) culture medium supplemented with 20% inactivated fetal bovine serum and blood-NNN medium (bacteriological agar with defibrinated rabbit blood), for approximately 45 days. Thus, it was possible to obtain a large enough volume for culture aliquots that were then employed in the isoenzyme analysis using the MLEE technique and in the molecular analysis, the latter of which occurred after the extraction of the genomic DNA.

### DNA extraction

Total DNA obtained from the clinical samples and the genomic DNA of the *L. infantum* cultures were extracted using the DNA QIAamp DNA Mini Kit (QIAGEN GmbH, Hilden, Germany), according to the manufacturer’s instructions. Negative controls for the DNA extraction process were used in all assays. The DNA extraction yield was determined by reading the absorbance at 260 nm in a Nanodrop ND-1000 (Thermo Fisher Scientific, Wilmington, DE, USA) spectrophotometer. The A_260_/A_280_ absorbance ratio was assessed to verify the purity of the obtained DNA.

### Polymerase chain reaction

In this study, two genomic targets from *L. infantum* were used for the PCR, namely, the kinetoplast DNA (kDNA) minicircle region and the *gp63* glycoprotein-encoding gene.

The specific reaction for the kDNA minicircle region was performed according to Cortes *et al*. [32], with some modifications. The primers used were MC1 (5’-GTTAGCCGATGGTGGTCTTG-3’) and MC2 (5’-CACCCATTTTTCCGATTTTG-3’), which amplify a sequence of 447 base pairs (bp). The reaction was prepared in a final volume of 100 μL, containing 5–10 μL of DNA (enough volume to contain 50 ng of DNA), 3 U of Platinum *Taq* DNA Polymerase (Invitrogen, São Paulo, Brazil), 1X PCR buffer (200 mMTris-HCl, pH 8.4, 500 mMKCl; Invitrogen), 0.6 μM each primer, 2 mM MgCl_2_ and 0.4 mMdNTPs (Promega, Madison, WI, USA). The thermal cycling program consisted of an initial step of 2 minutes at 94°C, followed by 35 cycles at 94°C for 20 seconds, 58°C for 20 seconds and 72°C for 30 seconds. A final extension step at 72°C for 5 minutes was also included.

The specific PCR for the *gp63* glycoprotein-encoding gene was performed according to Guerbouj *et al*. [26], with some modifications. The primers used were SG1 (5’-GTCTCCACCGAGGACCTCACCGA-3’) and SG2 (5’-TGATGTAGCCGCCCTCCTCGAAG-3’), which amplify a 1330-bp sequence. The reaction was prepared in a final volume of 40 μL, containing approximately 4 μL of DNA (enough volume to contain 30 ng of DNA), 1.5 U of Platinum *Taq* DNA Polymerase (Invitrogen), 1X PCR buffer (200 mMTris-HCl, pH 8.4, 500 mMKCl; Invitrogen), 0.6 μM of each primer, 1.5 mM MgCl_2_ and 0.2 mMdNTPs (Promega). The thermal cycling program consisted of an initial step at 94°C for 4 minutes and 35 cycles at 94°C for 1 minute, 67°C for 1 minute and 72°C for 1 minute, as well as a final extension step at 72°C for 8 minutes.

The amplification products were visualized in a 6% polyacrylamide gel after staining with 0.2% silver nitrate.

All assays included negative PCR controls (PCR mix without DNA), negative DNA extraction controls (PCR mix with the negative DNA extraction control) and positive PCR controls (DNA extracted from *L. infantum* promastigotes from the MHOM/BR/2002/LPC-RPV reference sample).

### Restriction fragment length polymorphism

Five of the six main endonucleases used by Cortes *et al*. [28] (*Vsp*I, *Dde*I, *Bgl*II, *Hpa*II and *Rsa*I; Promega) were employed to digest the 447-bp kDNA fragment. Two endonucleases were used for the digestion of the 1330-bp fragment of the *gp63* glycoprotein-encoding gene; i.e., *Hin*cII (according to Guerbouj *et al*. [26], with some modifications) and *Taq*I (according to Quispe-Tintaya *et al*. [27], Botilde *et al*. [21], Seridi *et al*. [33], with some modifications) (Promega).

The digestion reactions were performed separately for each enzyme in a final volume of 20 μL. All reactions contained 15 μL of the amplification product, 5 U of restriction enzyme, 1X buffer specific for each enzyme and 1 μg of bovine serum albumin. All enzyme reactions, except for that of *Taq*I, were incubated at 37°C for 3 hours and 30 minutes, followed by 10 minutes at 65°C, as recommended by the manufacturer. The reactions with the *Taq*I enzyme were incubated at 65°C for 3 hours and 30 minutes.

The products obtained from the restriction enzyme reactions were visualized on a polyacrylamide gel after staining with 6% ethidium bromide (0.5 mg/mL) and were photographed in an L-Pix EX gel documentation system (Loccus Biotechnology, Cotia, SP, Brazil).

The reference strains (Table 2) were also subjected to PCR-RFLP for comparison with the analyzed samples.

**Table 2:** **Strains used for a reference pattern**

**Identification**	**Species**	**International code**
Reference strain #1 (Ref 1)	*Leishmania (Leishmania) infantum*	MHOM/BR/2002/LPC-RPV
Reference strain #2 (Ref 2)	*Leishmania (Leishmania) infantum*	MHOM/BR/1974/PP75
Reference strain #3 (Ref 3)	*Leishmania (Leishmania) donovani*	MHOM/ET/1967/HU3
Reference strain #4 (Ref 4)	*Leishmania (Leishmania) amazonensis*	IFLA/BR/1967/PH-8
Reference strain #5 (Ref 5)	*Leishmania (Viannia) braziliensis*	MHOM/BR/75/M2903

The gel images were analyzed with the GelAnalyzer 2010 software (available at http://www.gelanalyzer.com). To more accurately determine the length of the fragments, a sample of each group was randomly selected (n = 7) and submitted to capillary electrophoresis in the Bioanalyzer 2100 device (Agilent Technologies GmbH, Waldbronn, Germany).

### Analysis of nucleotide sequences deposited in GenBank and subsequent in silico analysis in the RestrictionMapper program

The BLAST algorithm (http://blast.ncbi.nlm.nih.gov/Blast.cgi) was used to search for specific nucleotide sequences corresponding to the kDNA and *gp63* gene targets. For this purpose, the primer sequences MC1 and MC2 were used in the search, as were the previously described SG1 and SG2 primers. Only sequences that met the quality parameter with a high degree of identity (approximately 100%) with the primers and the *Leishmania* organism were selected. Seven sequences for the kDNA target and five sequences for the *gp63* target were analyzed. The sequences selected from GenBank originate from *L. infantum* or *L. donovani* samples isolated in different African and Central American countries, as well as in European countries (Table 3).

**Table 3:** **Nucleotide sequences selected from GenBank**

**Target**	**Identification**	**Species**	**Accession no./Country of origin**
kDNA	GenBank1	*L. infantum*	AF169140.1/Tunisia
	GenBank2	*L. infantum*	AF169133.1/Algeria
	GenBank3	*L. infantum*	AF103741.1/Tunisia
	GenBank4	*L. infantum*	AF169131.1/Portugal
	GenBank5	*L. infantum*	AF103740.1/United Kingdom^a^
	GenBank6	*L. donovani*	AJ010077.2/Sudan
	GenBank7	*L. chagasi*	AF169137.1/Panama
*gp63*	GenBank8	*L. infantum*	Y08156.1/France
	GenBank9	*L. infantum*	U48798.1/Spain
	GenBank10	*L. donovani*	AJ495003.1/Kenya
	GenBank11	*L. donovani*	AJ495006.1/Iraq
	GenBank12	*L. donovani*	AJ495004.1/China

^a^For this sequence of nucleotides, the patient was most probably infected in Spain (associated paper PUBMED: 10413058).

Subsequently, these nucleotide sequences were analyzed through *in silico* digestion in the RestrictionMapper version program (available at http://www.restrictionmapper.org), with the same enzyme panel as that used in the laboratory experiments. After restriction, the program generated the exact length of the obtained fragments. These sequences were compared to the study samples to infer phylogenetic relationships.

### *Phylogenetic analysis* for the *kDNA minicircle region*

**Figure 1:**
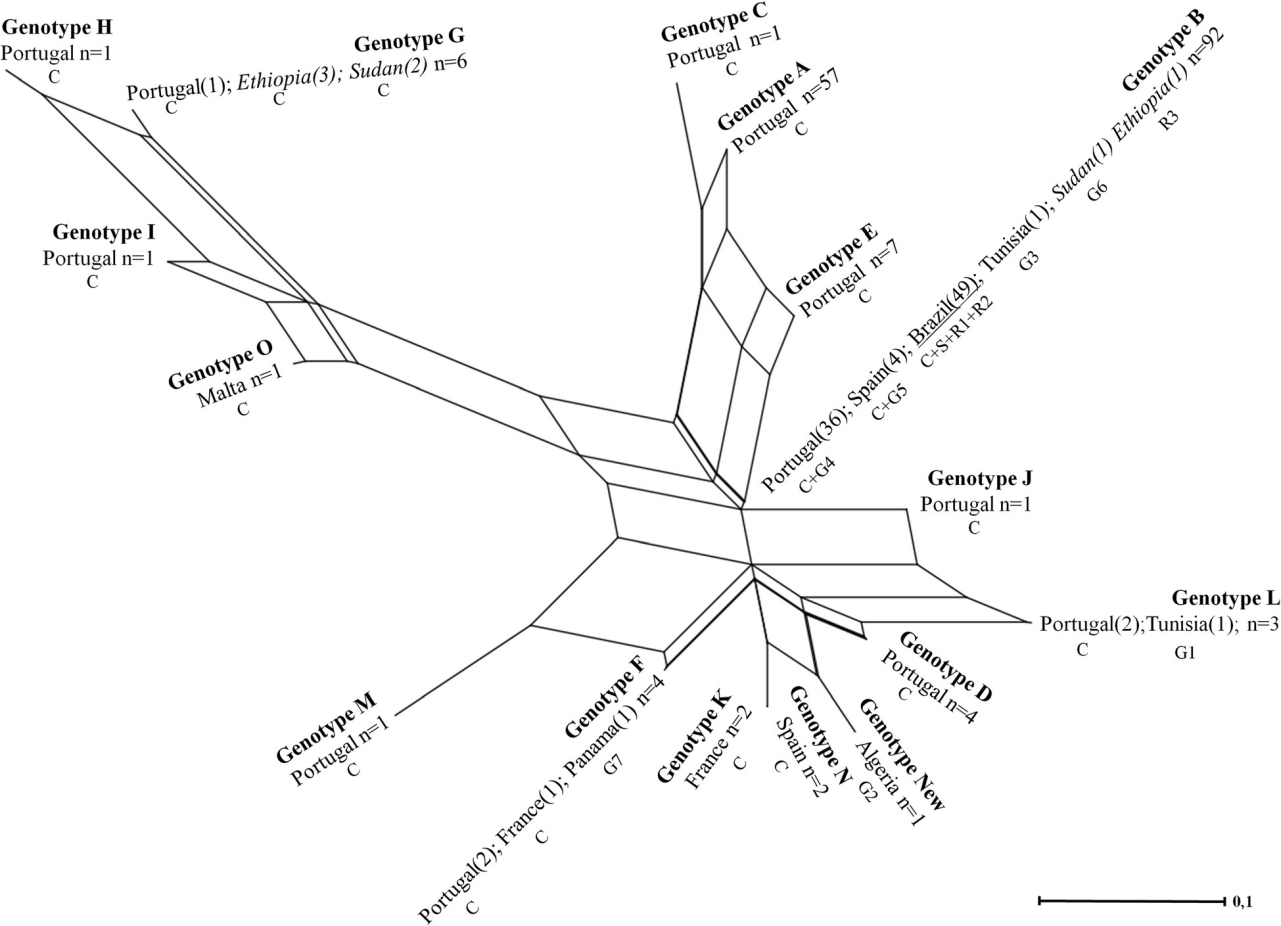
NeighborNet phylogenetic network from data of kDNA PCR-RFLP of Leishmania infantum/L. donovani isolates from samples. *Leishmania donovani* samples are italicized. Samples from Brazil are underlined. C, samples from Cortes *et al*. [28]; G, nucleotide sequences from GenBank; R, reference strain; S, samples of this study.

To analyze the phylogenetic relationships, the restriction patterns resulting from the cleavage of the PCR products by the endonucleases were used to determine profiles or genotypes and were interpreted through qualitative analysis. The 45 samples of this study were phylogenetically compared to the 129 described by Cortes *et al.* [28] (classified as genotypes A to O) as well as to the seven samples previously selected in GenBank and three reference strains (Ref 1, 2 and 3) forming a database with a total of 184 samples. The genotypes identified in this study were named according to Cortes *et al*. [28]. Restriction patterns from all samples were inserted into a binary matrix, with the fragments coded as present (1) or absent (0). The SplitsTree4 software was used for the construction of a crosslinked network (Figure 1) [34,35].

The crosslinked network is more appropriate than the bifurcated phylogenetic tree to describe the complex relationship populations because it shows conflicts in a data set, without prior assumptions of the causes of these conflicts [36].

### Ethical considerations

This study was approved by the Research Ethics Committee of the Federal University of Minas Gerais, in a collaborative study with Centro de Pesquisas René Rachou/FIOCRUZ and Federal University of OuroPreto (CAEE-03904112.0.0000.5149). In addition, the canine samples collection was approved by the Committees of Ethics in Animal Experimentation of the Universidade Federal de Ouro Preto protocol no. 083/2007), of the Universidade Federal de Minas Gerais (protocol no. 020/2007), and of the City Council of Belo Horizonte (protocol no. 001/2008). All procedures in dogs in this study were according to the guidelines set by the Brazilian Animal Experimental Collage (COBEA), Federal Law number 11794.

## Results

All 45 DNA samples analyzed in this study showed PCR amplification of the kDNA gene fragment (447-bp), specific for the *Leishmania donovani* complex, and the *gp63* gene fragment (1330-bp), specific for trypanosomatids.

The PCR assay for the kDNA target showed a detection limit of 1 pg of genomic DNA (1:10 serial dilution) of the *L. infantum* reference sample (MHOM/BR/2002/LPC-RPV), while the detection limit was 10.42 fg for the *gp63* target.

The PCR-RFLP analysis of both targets (i.e., kDNA and *gp63*) in the 45 DNA samples analyzed revealed the genetic homogeneity of *L. infantum*. Thus, for each analyzed endonuclease, a single enzyme restriction pattern was observed (Table 4). However, there was some variability between the nucleotide sequences obtained after searching GenBank (Table 3) and the subsequent *in silico* analysis in the RestrictionMapper program.

**Table 4:** **Restriction enzyme patterns obtained with PCR-RFLP**

**Targets**	**kDNA**					***gp63***	
Endonucleases	*Vsp*I^a^	*Dde*I^a^	*Bgl*II^a^	*Hpa*II^a^	*Rsa*I^a^	*Hinc*II^b^	*Taq*I
Fragments (bp)	161	319	447	410	253	1250	504
	152	100		37^c^	194	850	414
	134	28^c^				400	180
						80	84
							80
							29^c^
							22^c^
							20^c^

^a^Pattern I, according to Cortes *et al*.[28]; ^b^Pattern I, according to Guerbouj *et al*. [26]; ^c^Fragments lost during electrophoresis.

### PCR-RFLP for the kDNA minicircle region

After restriction enzyme analysis by PCR-RFLP of the kDNA minicircle region (447-bp), the following patterns were found in the 45 DNA analyzed samples: the *Vsp*I enzyme produced 134-, 152- and 161-bp fragments; the *Dde*I enzyme produced 28-, 100- and 319-bp fragments; and the *Bgl*II enzyme produced only one 447-bp fragment; that is, the fragment remained intact, with no specific restriction site for this enzyme in the sequence. These patterns were also observed in all seven nucleotide sequences for the kDNA target obtained after searching the GenBank (GenBank 1,2,3,4,5,6 and 7) and subsequent *in silico* analysis in the RestrictionMapper program and in the reference strains MHOM/BR/2002/LPC-RPV (Ref1), MHOM/BR/1974/PP75 (Ref2) and MHOM/ET/1967/HU3 (Ref3). All of these patterns for the analyzed enzymes are similar to that described by Cortes *et al*. [28], known as Pattern I.

For the enzyme *Hpa*II, 37- and 410-bp fragments were observed for all 45 DNA samples evaluated. This same pattern was observed in the reference strains: Ref1, Ref2 and Ref3, and in four nucleotide sequences for the kDNA target: GenBank 3, 4, 5 and 6. This pattern is known as Pattern I, according to Cortes *et al*. [28]. Three of the seven nucleotide sequences obtained after the GenBank search and subsequent *in silico* analysis in the RestrictionMapper program showed genetic variability: AF169140.1/GenBank1-kDNA (Tunisia/North Africa), AF169133.1/GenBank2-kDNA (Algeria/North Africa) and AF169137.1/Genbank7-kDNA (Panama/Central America). The first nucleotide sequence (AF169140.1/Genbank1-kDNA) produced 37-, 123- and 287-bp fragments, considered by Cortes *et al*. [28] as Pattern II for the *Hpa*II enzyme. The second nucleotide sequence (AF169133.1/GenBank2-kDNA) produced 123- and 324-bp fragments, not yet described in the literature. Finally, the last nucleotide sequence (AF169137.1/Genbank7-kDNA) produced 37-, 60- and 350-bp fragments, described by Cortes *et al*. [28] as Pattern IV.

The analysis using the *Rsa*I enzyme revealed a single pattern with 194- and 253-bp fragments for the 45 DNA samples analyzed in this study, as well as for the reference strains (Ref1, Ref2 and Ref3) and six nucleotide sequences: GenBank 2, 3, 4, 5, 6 and 7. This pattern was described as Pattern I, according to Cortes *et al*. [28]. The AF169140.1/GenBank1-kDNA nucleotide sequence exhibited variability, including 48-, 146- and 253-bp fragments, the same pattern described by Cortes *et al*. [28] as Pattern IV for the *Rsa*I enzyme.

### Phylogenetic analysis − minicircleregion kDNA

The phylogenetic analysis for samples tested by PCR-RFLP for the target kDNA showed that all 45 samples analyzed as well as reference samples 1 and 2 were within the Genotype B described by Cortes *et al*. [28] (Figure 1). The two samples of Brazilian origin used by Cortes *et al*. [28] were also included in the same genotype. Samples from Portugal (36), Spain (4), Tunisia (1) Sudan (1) and Ethiopia (1) were also included in this genotype. The latter two samples are *L.donovani*, GenBank6 and Ref3, respectively. The other samples are *L.infantum.*

It was observed that within the cluster in which the genotype B is inserted, samples of genotype A, C and E from Portugal were also included.

The other genotypes (D, F, G, H, I, J, K, L, M, N, O, and new) were located in other phylogenetic network clusters and include samples from different countries. The genotypes G and B were the only samples with another species, *L.donovani*. In genotype G, three of these samples were from Ethiopia and one from Sudan. A different genotype was identified, not described in the literature, and was named “New Genotype.” This was a sample obtained from GenBank (GenBank2) from Algeria.

### PCR-RFLP for the gp63 glycoprotein-encoding gene

Analysis with the *Hinc*II enzyme produced a single pattern with four fragments, 80-, 400-, 850- and 1250-bp, for the 45 DNA samples analyzed, the reference strains (Ref1, Ref2 and Ref 3) and all five nucleotide sequences: GenBank 8, 9, 10, 11 and 12 (Table 3). The 80- and 1250-bp bands were clearer and exhibited better resolution than the 400- and 850-bp bands. Pattern I was also described by Guerbouj *et al*. [26] for the *Hinc*II enzyme.

With the *Taq*I enzyme, only one pattern, Pattern I, could be identified, with five fragments: 80-, 84-, 180-, 414- and 504-bp. Several authors have used the PCR-RFLP technique to analyze the *gp63* glycoprotein-encoding gene using the restriction endonuclease *Taq*I; however, the fragment patterns produced were not clearly shown in those studies [21,27,33]. Thus, it was not possible to perform phylogenetic analysis for the target *gp63*.

Pattern I found in the 45 DNA samples analyzed in this study (80-, 84-, 180-, 414- and 504-bp fragments) can be compared to the nucleotide sequences for the *gp63* target obtained after searching GenBank and subsequent *in silico* analysis in the RestrictionMapper program. The same pattern was obtained for the nucleotide sequence U48798.1/GenBank9-*gp63*. This sequence also exhibited 20-, 22- and 29-bp bands that could not be visualized in the (6%) polyacrylamide gels analyzed in this study, due to the low molecular weight and losses during electrophoresis.

## Discussion

The present study evaluated the intraspecific genetic variability of *L. infantum* in human and canine VL cases from BHMA through the use of PCR-RFLP and different target genes: the *gp63* glycoprotein-encoding gene and minicircle region of kDNA. The analysis showed genotypic homogeneity in the studied samples, with regard to all tested endonucleases for the target genes. However, some nucleotide sequences from GenBank and from *L. infantum* or *L. donovani* specimens isolated in different African, Central American, and European countries showed genotypic variability. One of the strains from GenBank (GenBank2) demonstrated a band profile not described in the literature, which we named New Genotype in this work. These findings show that the methodology chosen for this study was effective for evaluating the intraspecific genetic variability of *L. infantum*, considering its relationship with geographical origin in different countries, which gives even greater strength to the results obtained with the Brazilian samples.

In addition to the 45 *L. infantum* DNA samples evaluated by PCR-RFLP, two other DNA samples extracted from *L. infantum* cultures of two VL-symptomatic patient samples were included in the study by convenience sampling (data not shown). These samples originated from the Minas Novas and Aimorés municipalities, which are approximately 516 km and 456 km from Belo Horizonte, respectively. Even with the inclusion of those samples from municipalities that are not part of the BHMA, genetic homogeneity was consistent among the targets evaluated in the study samples.

Other studies conducted in Brazil, in general, also found low genetic variability among the studied samples [12,15,16,18,29]. Specifically, Segatto *et al*. [14] evaluated the genetic variability of *L. infantum* in samples from different regions of Brazil and also found no differences between human and dog samples. Chicharro *et al*. [37] obtained similar results using the RAPD and intergenic region typing of the ribosomal RNA internal transcribed spacer region techniques, finding no genotypic differences between *Leishmania* isolates from dogs and humans of Majorca Island, Spain.

It is important to note that this is the first study that simultaneously evaluates two targets (kDNA and *gp63*) using both samples from humans (children and adults with or without *Leishmania*/HIV co-infection) and from dogs with different clinical forms of VL (symptomatic, oligosymptomatic and asymptomatic) to monitor *L. infantum* population spread at RMBH.

The kDNA was one of the selected targets because minicircles are useful targets for evaluating genetic variability due to their involvement in a rapid response to different environmental conditions and physiological stress situations [11,28]. This characteristic most likely interferes with the adaptability of the parasite, which may acquire selective advantages in accordance with the most prevalent minicircle class within the kDNA network [11,38]. The other selected target was the *gp63* glycoprotein because it is a very important protein in the virulence of the parasite, and the gene is under constant selective pressure in the phase of the parasite’s adaptation; therefore, the protein is a promising target for the study of genetic variability in *Leishmania* [26,39].

Several molecular approaches have been used to study genetic variability of parasites of the genus *Leishmania*, but in most studies, MLMT [13] and RFLP [11] have proven to be the best tools for analyzing population genetics and epidemiological data. In this study, PCR-RFLP was used because, in addition to the technique’s high sensitivity, ease of execution and good reproducibility of the results obtained, it allows a refined distinction between parasite strains through the identification of polymorphisms in the specific restriction sites for the evaluated endonucleases [28,33].

Phylogenetic analyses indicated great genetic variability among the samples from Portugal because these were present in almost all genotypes found. However, a greater number of Portuguese samples were concentrated in two genotypes: A (57 samples) and B (36 samples). The B genotype also accounted for all Brazilian samples (49) analyzed: 45 samples from the metropolitan region of Belo Horizonte, two samples of Cortes *et al.* [28] and two samples used as reference *L.infantum* (Ref1 and Ref2). These data indicate that infection by *L.infantum* (genotype B) is common in both Brazil and Portugal. The results found in the present study corroborate the hypothesis of the introduction of *L. infantum* in South America, through the great sea voyages that took place approximately 500 years ago [4,18,40]. Those studies concluded that 500 years is a relatively short time to generate genetic variability, especially in Brazil, where VL has one main vector (*L. longipalpis*) and the dog (*Canis familiaris*) as the main reservoir. However, to confirm this hypothesis, genotyping samples from other geographical regions of Brazil is necessary in view of the disease’s large territorial range.

Moreover, prior studies also took into consideration that the prevalent reproduction mode of species of the genus *Leishmania* is clonal; i.e., their genotypes can be used as reliable epidemiological markers, thus facilitating the study of the dispersal of this parasite [41,42]. Although genetic recombination has been recorded by some authors for the genus *Leishmania* [43-45], this phenomenon is still considered a rare event.

## Conclusion

In our work, we showed that no genetic variability occurred between the different evaluated groups, considering the different types of hosts (dogs and humans), as well as the various clinical forms of VL in dogs and distinct aspects related to age, sex and the presence or absence of *Leishmania*/HIV co-infection in humans. Our results suggest that only one *L. infantum* strain is circulating in human and canine populations in BHMA. However, other studies should be done in BHMA with a larger number of different host samples (human, dogs and phlebotomine sand fly), and additional molecular techniques should be used to provide a more complete picture of the *L. infantum* genetic variability in the region and to complement our results.

## Abbreviations

BHMA: Belo Horizonte Metropolitan Area
kDNA: kinetoplast DNA
MLEE: Multi-locus enzyme electrophoresis
MLMT: Multilocus microsatellite typing
PCR: Polymerase chain reaction
RFLP: Restriction fragment length polymorphism
SSR: Simple sequence repeat
VL: Visceral leishmaniasis
